# Exploring land cover change after prolonged droughts at the global level

**DOI:** 10.1038/s41598-025-14713-6

**Published:** 2025-08-05

**Authors:** Felicia Engman, Ester Kortekaas, Luigia Brandimarte, Maurizio Mazzoleni

**Affiliations:** 1 Department of Sustainable Development, Environmental Science and Engineering, Royal Institute of Technology, KTH, Stockholm, Sweden; 2 Institute for Environmental Studies, Vrije Universiteit Amsterdam, Amsterdam, The Netherlands; 3 Department of Global Public Health, Karolinska Institutet, Stockholm, Sweden

**Keywords:** Drought, Land cover change, Global analysis, Income level, Climate zones, Natural hazards, Hydrology

## Abstract

Anthropogenic climate change is increasing the frequency and severity of droughts, which accelerate land degradation, harm ecosystems, and threaten water security and livelihoods. However, the global impact of prolonged droughts on land cover changes remains poorly studied. This research analyses precipitation data to identify drought conditions and compares them with land cover datasets. The analysis explored three analytical dimensions, over the period 1992–2021: country level, income level, and climatic level. Results show a strong connection between droughts and land cover changes, with low-income countries experiencing the most significant changes. In drought-prone areas, trees, cropland, and sparse vegetation are the most impacted, with correlations stronger than in unaffected regions. This study provides valuable insights into the complex relationship between global drought events and land cover changes, varying across regions and economic contexts, and highlights the mitigating role of proactive strategies such as sustainable land management and adaptation measures.

**Supplementary Information:**

The online version contains supplementary material available at 10.1038/s41598-025-14713-6.

## Introduction

Increasing global temperatures have altered the frequency, duration, and spatial extent of droughts over the last decades^1^. Anthropogenic climate changes are anticipated to exacerbate the severity of drought events by altering the spatial and temporal patterns of precipitation and temperature^2–4^. Future drought impacts are expected to increase due to population growth, urbanisation, socioeconomic development, and other human activities, which drive higher water consumption rates^5^.

Land use and land cover management can significantly affect drought occurrence and drought impacts. Unsustainable land-use practices, such as deforestation, intensive agriculture, and urban sprawl amplify the occurrence and severity of droughts by disrupting hydrological cycles, depleting groundwater and increasing water demand. The conversion of natural vegetation into croplands often reduces soil moisture retention and depletes groundwater reserves, leading to heightened susceptibility to drought^6^. Orimoloye et al.^7^ revealed that cultivated agricultural lands and barren surfaces are particularly vulnerable during extreme drought events, as in 2015 in South Africa. Similarly, deforestation can significantly alter hydrological cycles, reducing the land’s ability to retain water and regulate local climates. Deforested lands experience higher rates of evaporation and surface runoff, leading to decreased groundwater recharge and exacerbating drought impacts^8–10^. This issue is particularly concerning in tropical areas such as the Amazon Basin, where widespread deforestation has been linked to regional drying trends and the increased frequency of extreme droughts^11^. Shrub- and grassland are ​particularly vulnerable to drought according to a global study^12^with productivity declines exceeding 35%. This highlights their heightened sensitivity to water stress due to shallow root systems and limited water storage ​​capacity. ​Moreover, drought has been shown to negatively affect grassland biodiversity by causing local extinction of species^13^. ​In arid and semi-arid areas, droughts have particularly severe impacts due to already limited water availability. The loss of vegetation leads to soil erosion, crusting, and salinization, which further reduce water infiltration and storage. As organic matter declines, fertility and biological activity decrease. This creates a self-reinforcing cycle of land degradation known as desertification^14^. The rapid expansion of urban areas also influences drought occurrence and severity. Urbanisation increases impervious surfaces reducing the capacity for groundwater recharge and increasing runoff during rainfall events. Urban regions experienced severe water stress due to the combined effects of land-use change and increased water consumption driven by population growth^5,15^. Urbanisation also amplifies the urban heat island effect, which accelerates water loss from soils and vegetation, further aggravating drought impacts^16^placing a disproportionate burden on vulnerable communities^17^. On the other hand, effective land management can mitigate drought impacts. Practices such as sustainable agriculture, reforestation, and the preservation of natural landscapes enhance soil moisture retention and improve the resilience of ecosystems to drought^18^.

While society and human activities can shape drought impacts, drought can have a significant effect on a number of human activities. Agriculture, in particular, is highly vulnerable to drought, as drier and hotter climates are likely to result in crop damage, reduced arable land, and diminished access to irrigation water. These impacts exacerbate socio-economic challenges, such as food and water insecurity, threats to livelihoods, and heightened health risks^19^. In addition, the increasing frequency and severity of droughts are expected to intensify health-related impacts, including waterborne and vector-borne diseases, malnutrition, and cardiovascular and respiratory illnesses linked to drought-induced air quality degradation^20,21^. Ecosystems are similarly vulnerable to drought, as reduced water availability can hinder plant growth, disrupt natural water flow, degrade water quality, and threaten both plant and animal species^22^. Forests, in particular, depend on consistent moisture and are highly sensitive to drought. Extended dry periods can cause tree mortality, canopy thinning, and shifts toward less water-demanding species - altering forest structure and function^23^. Grasslands and agricultural lands face similar challenges, including reduced plant growth and increased soil exposure, which can lead to desertification, as observed in the Sahel region^24^.

Satellite data consistently show declines in vegetation cover during droughts across multiple biomes^25^. This loss of plant cover leaves soil vulnerable to erosion, depletes organic matter, and disrupts soil structure, reducing long-term land productivity^26^. Drought also reshapes plant communities, favoring drought-tolerant species, which impact biodiversity and ecosystem services. In arid and semi-arid areas, this often results in the replacement of perennial plants with annual grasses or shrubs^27,28^. In some cases, drought can serve as tipping points, leading to permanent landscape changes. In the Amazon, for example, repeated droughts combined with deforestation have triggered a shift from rainforest to savanna-like ecosystems^29^.

The influence of drought on land cover change remains largely underexplored, despite growing evidence of its ecological impacts. Most existing research has focused on how land cover affects drought, rather than the reverse. Where drought-driven changes have been studied, they are often limited to specific regions or land cover types. For instance, agricultural and aquaculture shifts have been observed in coastal Vietnam^30^and a transition from woodlands to mixed farming has occurred in Ethiopia’s Rift Valley^31^. Other studies have examined land–atmosphere feedbacks across Europe^16^the effects of drought on vegetation productivity at the global scale^32^and large-scale land use change driven by trade and economic forces^33^.

To our knowledge, no global-scale study has directly assessed how drought events influence structural transitions in land cover. Our study addresses this by providing a systematic, global analysis of drought impacts on land cover, across different income groups and climatic regions. Specifically, it investigates how prolonged drought periods (12 months) affect land cover change at the global scale. The study focuses on the following three research objectives:

- Assess the relationship between drought occurrence and changes in land cover.
- Compare land cover changes within and outside drought areas.
- Assess how different land cover types are affected by prolonged drought periods.

This is achieved by exploiting freely available global datasets of precipitation to calculate the Standardized Precipitation Index (SPI) and assess drought conditions. The Copernicus dataset was selected to represent the annual spatial distribution of different land cover types over the period 1992–2022, at the global level. Assuming a specific year of interest, drought areas were identified and land cover change occurring in the year after the prolonged drought was assessed. Detailed information on data and analysis can be found in the methods session.

## Results

### Global trends of land cover change after drought periods

**Fig. 1:**
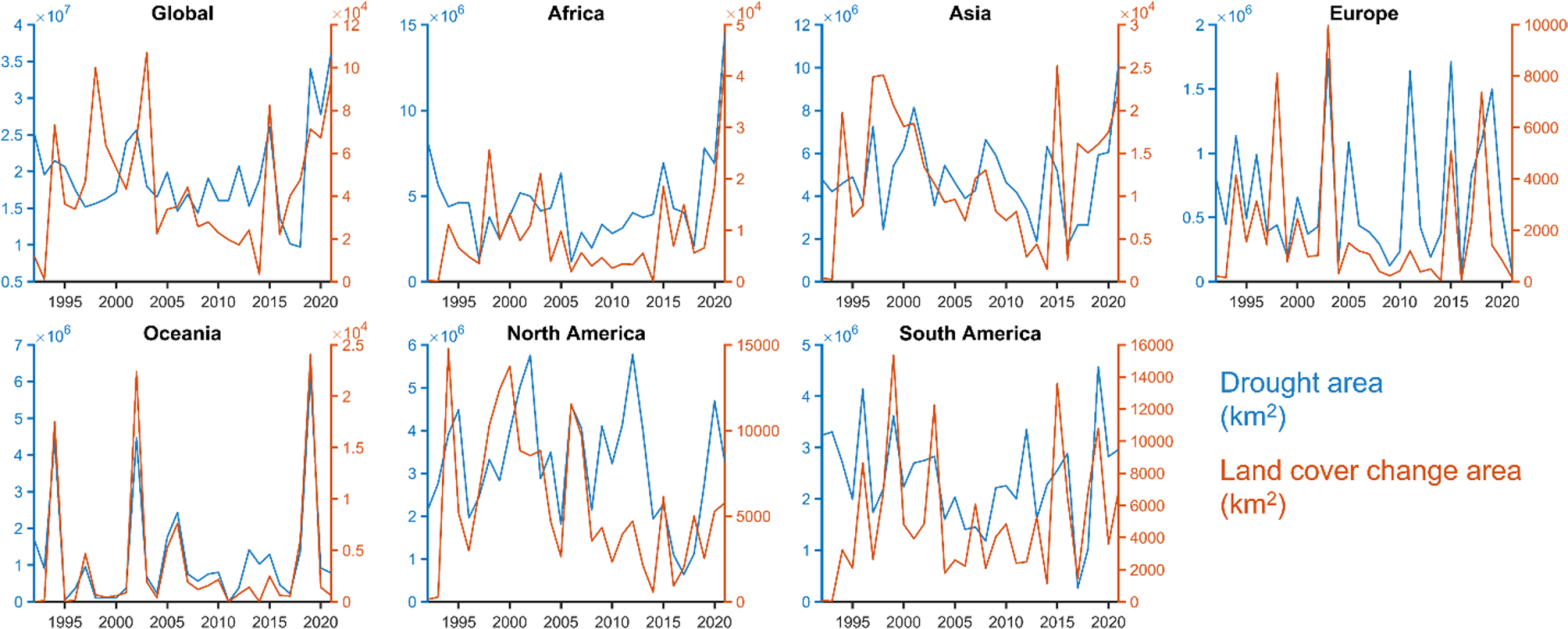
Global temporal variation of drought and land cover. Time series of drought area (left axis) and land cover change area (right axis) globally in the period 1992–2021.

This analysis explores the trend of land cover changes after a prolonged drought period at country level, worldwide. At the global level (Fig. 1), the period between 2004 and 2014 appears characterised by relatively low land cover changes, coinciding with a generally stable period of drought impacts. Conversely, a significant increase in land cover change is evident in 1994–2003. A comparable pattern emerged around 2015, followed by a continuously increasing trend after 2016. Comparatively, global drought events consistently range between 10 and 20 million km^2^with pronounced increases observed in 2015 and an increasing trend from 2018 onwards. In Oceania, as corroborated by the Australian Government’s Bureau of Meteorology, with the millennium drought, one can observe drier conditions during late 2017–2019 and extensive bushfires during 2019–2020 (Fig. 1). The annual drought area in Africa is relatively stable, around 4 million km^2^with a significant drought event in the Horn of Africa between 2020 and 2022, linked to the most severe rainfall deficit on record^34^ leading to a drought area of about 15 million km^2^ and a similar land cover change increase of up to 40 thousand km^2^.

In Asia, a noticeable increase in drought area changes occurred during 1993, 1996–2000, and in 2014, with high land cover changes in 1996, 1997, and 2016. A strong link between drought and land cover change areas is found in Europe: major drought events identified in 2003, 2015, and 2018 correspond with drought events found in the research literature^35,36^ and changes in land cover largely follow such drought events. However, a low alignment between drought and land cover changes in Europe in 2011 is observed. North and South America exhibit a moderately strong relationship between drought events and land cover change. Over the past three decades, North America has experienced several significant drought events, notably the most severe occurring during 1993-1994^37^ and 2004-2008^38^, patterns that are further supported by existing literature. Substantial land cover change occurred following these drought events. However, while North America shows high drought area values in 2008–2012, low land cover change values are experienced in the same period. Conversely, South America experienced a high land cover change in 2003, despite the relatively low drought area, suggesting that other activities may have contributed to the change in land cover during that period. Venezuela and the southeastern part of Brazil suffered droughts 2014-2016^39^. In Brazil, a subsequent drought event took place in 2018, which later extended to Paraguay, Bolivia, and northern Argentina in the following years. For all the drought events highlighted in the literature, drought peaks can been seen in Fig. 1, followed by significant land cover changes.

**Fig. 2:**
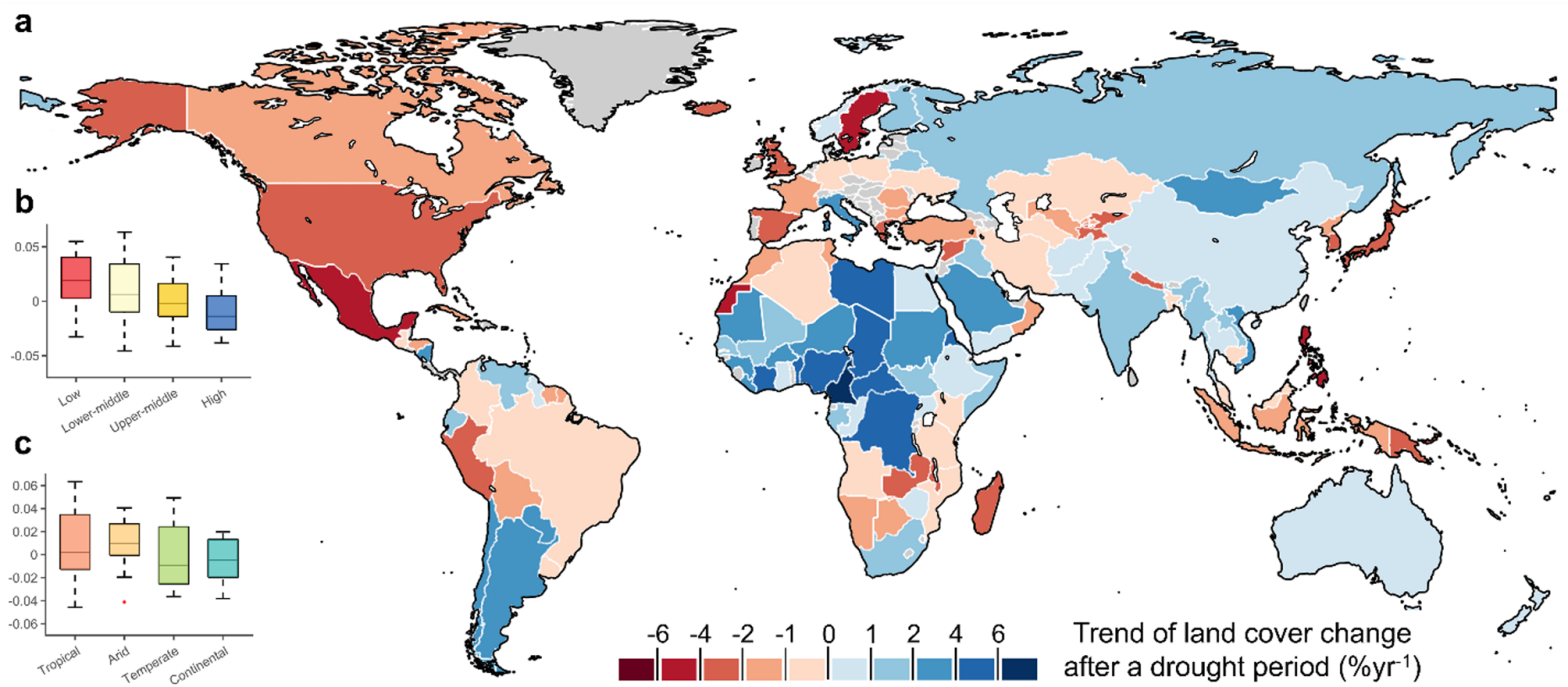
Global trends of normalised land cover change after a drought. **a** Trends in drought-affected areas after a drought event at country level. Classification of trends in land cover change by country income level (**b**) and climate areas (**c**). Matlab r2023a and shapefile of world countries borders retrieved from https://www.geoboundaries.org/ were used to produce this map.

We then calculated the normalized land cover change trend at the country level in drought-affected areas (Fig. 2). Z-score normalisation was performed (see methods) to better compare results across countries of different sizes. We found a visible global pattern, with a generally positive trend in land cover change within drought areas. Africa, Asia, and part of South America show a positive trend in land cover change, while North America, Europe, and part of Oceania show a negative trend. The highest trend values are found in North and Central Africa, while the lowest are in Mexico, Sweden, and Japan. Our findings show that low-income countries are the ones experiencing higher trends of change in land cover after prolonged droughts, with high-income countries showing the lowest trends. In particular, upper-middle and high-income countries have a negative land cover change trend, meaning that over time, land cover tended not to change and remained stable after drought occurrence. This can be linked to the fact that in high-income countries land cover change is driven by other socio-economic factors rather than drought occurrence. Similarly, high trends are found in tropical areas, while lower values are seen in continental climate areas.

### Correlation analysis between changes in drought and non-drought areas

The previous results provided valuable insights into the global trends of land cover change in a drought area after a drought event. However, these trends could be general trends unrelated to the drought occurrence. For this reason, we correlated changes in land cover in drought-affected areas with changes in non-drought areas. This comparison aims at providing a clearer understanding of the influence of drought on land cover change.

**Fig. 3:**
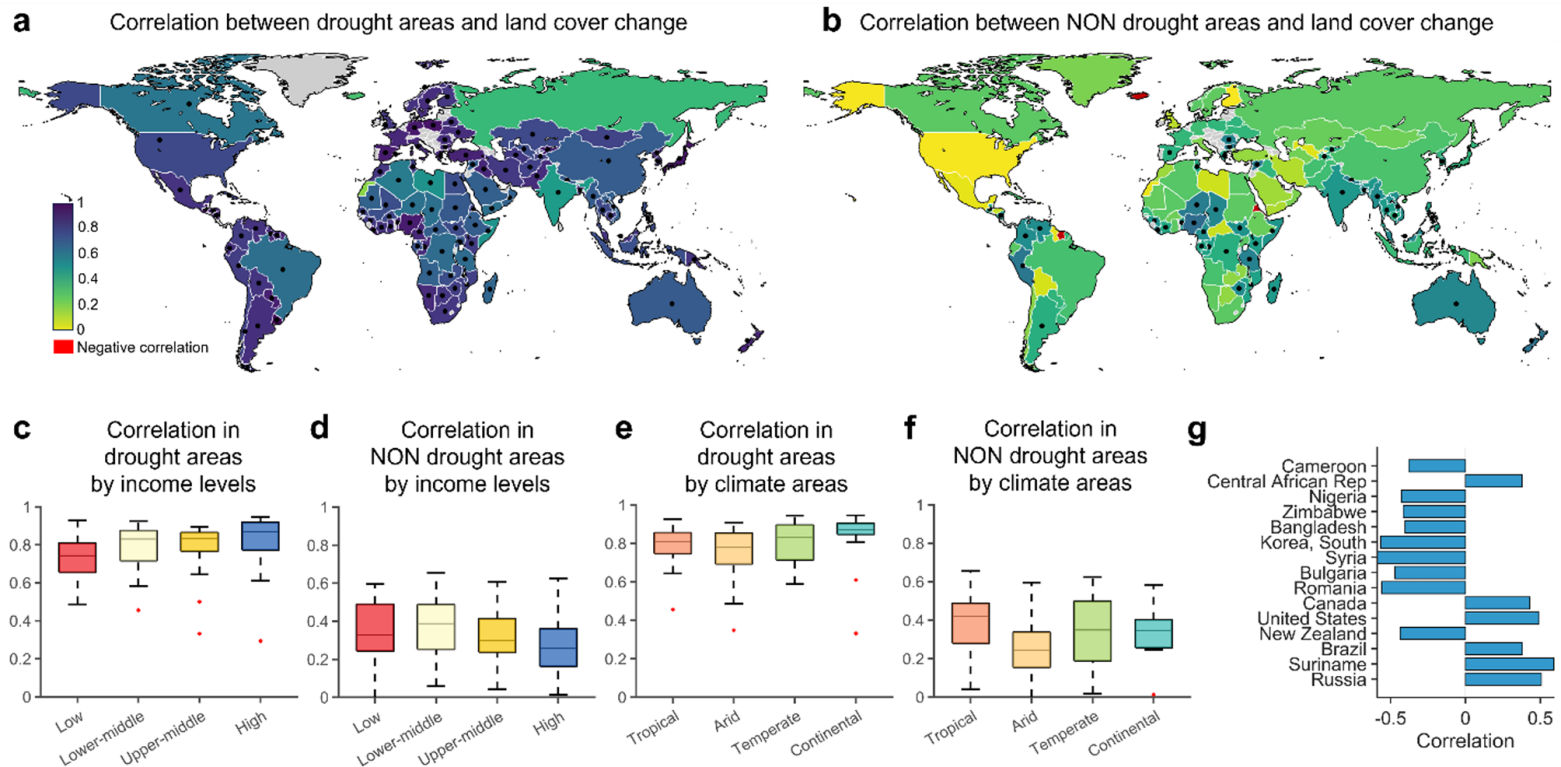
Correlation between drought areas and land cover change. Spatial distribution of the correlation (with black dots within the maps representing the statistical significance level *p* < 0.05) in drought areas (**a**) and non-drought areas (**b**). Classification of correlation values per income level (**c** and **d**) and climate areas (**e** and **f**). Values with statistically significant correlation (*p* < 0.05) between land cover changes in drought and non-drought areas (**g**). Matlab r2023a and shapefile of world countries borders retrieved from https://www.geoboundaries.org/ were used to produce this map.

Overall, we found a stronger correlation between land cover change in drought areas than in non-drought areas (Fig. 3a and b). This result strengthens the link between land cover changes and drought occurrence. The correlation between drought and land cover change exhibits significant variation across world regions. Higher correlation values in drought areas are found in Europe, South America, and West Asia. On the other hand, the lowest correlation in non-drought regions are found in North America and negative correlation in just 5 countries out of 109. Moreover, correlation values in drought areas are for the majority statistically significant (*p* < 0.05), while in non-drought regions only a few countries show substantial results.

Higher correlation values between drought area and land cover change are found in high-income countries, with lower values (around 0.8) observed in low-income countries (Fig. 3c and d). On the other hand, higher values are found in low-income countries when focusing on correlation in non-drought areas, with lower values in high-income countries. This means that while the trend of land cover change is higher in low-income countries than in high-income countries, this can be linked to other socioeconomic factors affecting land cover change rather than drought occurrence, as the correlation is lower. Similar patterns are found across the different climate areas (Fig. 3e and f). Finally, we found that only 15 countries showed statistically significant results when assessing the correlation between land cover change in drought areas and non-drought areas (Fig. 3g). This is in line with the results in Fig. 3a and b.

### Change of land cover type after a drought event

While the previous results provide valuable insights into the land cover change in drought and non-drought areas, they do not distinguish among land cover types. This third analysis explores how different land cover types changed after a prolonged drought. To compare changes in land cover types with different spatial extents, we assessed the coefficient of variation as the ratio between standard deviation and mean of land cover change over 1992–2021, for a given country.

**Fig. 4:**
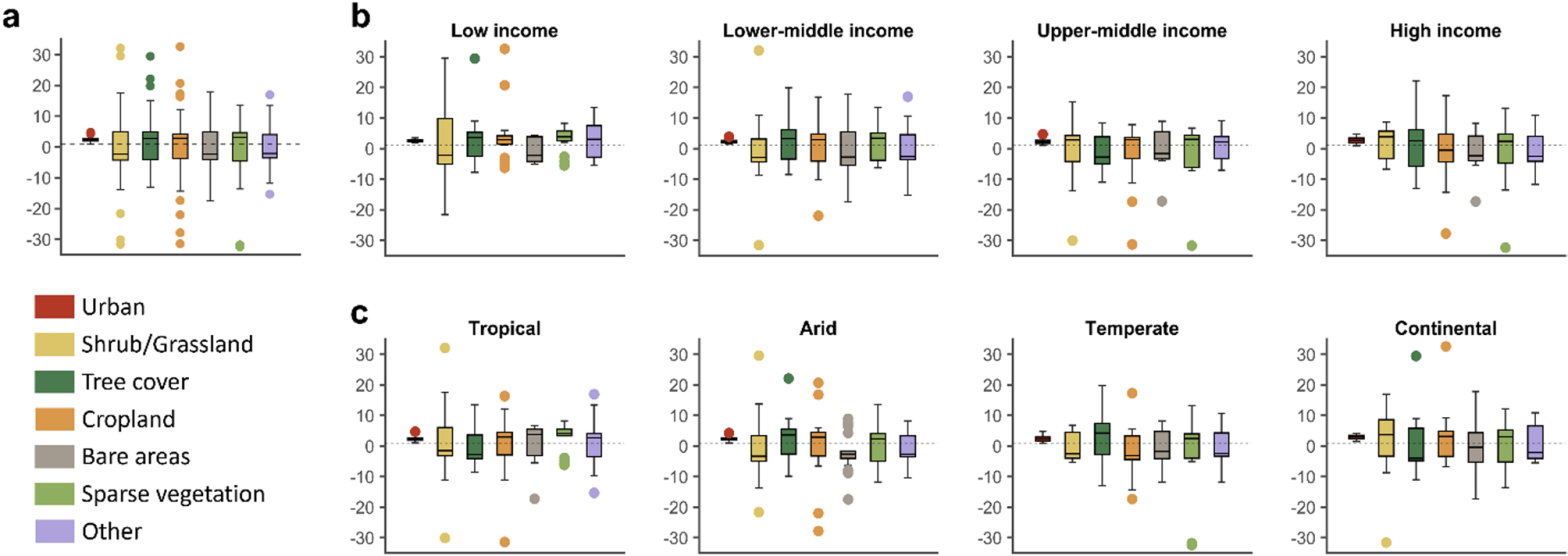
Land cover change variation after drought. Coefficient of variation of different land cover types after prolonged drought periods assessed worldwide (**a**) and classified by income levels (**b**) and climate areas (**c**).

Our findings reveal that shrub/grassland, bare areas, and other land cover types tend to decrease after a prolonged drought, while tree cover, cropland, sparse vegetation, and urban areas increase, at the global level (Fig. 4a).

In particular, shrub/grassland is the land cover with the highest variation across low-income countries, with a median negative variation (Fig. 4b). Land cover types that, on average, show a positive coefficient of variation in low-income countries after a drought are tree, cropland, and sparse vegetation. Tree cover type shows a high positive variation in lower-middle and high-income countries, with negative variation in upper-middle countries. Cropland cover type increases (positive mean coefficient of variation) across low- and middle-income countries after a drought period and decreases in high-income countries. Similar results are found for urban and sparse vegetation areas, with positive variation across all the countries. On the other hand, bare area tends to decrease across countries.

We found that shrub/grassland is reduced in tropical, arid, and temperate climates, while it increases in continental climate (Fig. 4c). On the other hand, tree cover reduces in continental and tropical climates, while increases in arid and temperate after prolonged droughts. Similarly to the income classification, urban and sparse vegetation increase across different climates.

**Fig. 5:**
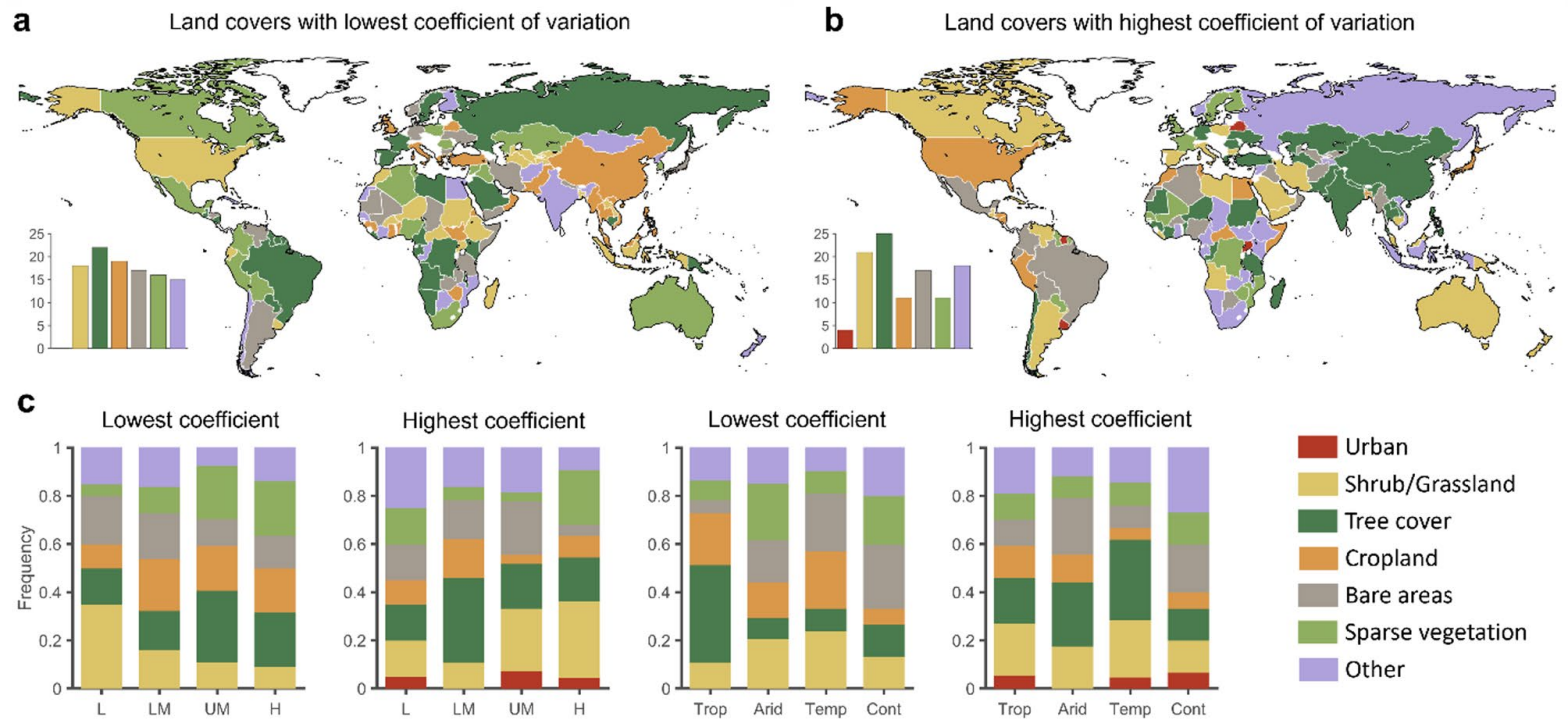
Spatial representation of extreme land cover change variation after drought. Countries with the lowest (**a**) and highest (**b**) coefficient of variation for different land cover types, classified by income level and climate areas (**c**). Matlab r2023a and shapefile of world countries borders retrieved from https://www.geoboundaries.org/ were used to produce this map.

We finally assessed which land cover type had the lowest and highest coefficient of variation across income levels and climates. Overall, tree cover is the most predominant one subject to changes, followed by shrub/grassland (Fig. 5a and b). Tree cover shows the highest coefficient of variation in Asia and arid and temperate climates (Fig. 5c). Countries in which tree cover is predominant regarding the lowest coefficient of variation are located in Africa and South America. Cropland and shrub/grassland are characterised by the highest coefficient of variation in countries in North America, South America, East Asia, and Oceania.

Only two countries have urban cover with the lowest variation, while six countries have the highest variation. These countries are located in continental climate areas and have upper-middle to high incomes. Shrub/grassland and cropland are the cover types with the lowest coefficient of variation in low and lower-middle income countries. In contrast, tree cover is the predominant one with the lowest coefficient of variation in upper-middle and high-income countries (Fig. 5c). On the other hand, shrub/grassland is the predominant cover type with the highest coefficient of variation in upper-middle and high-income countries. In contrast, trees and other cover types are predominant in low and lower-middle countries.

## Discussions

We analyzed high-resolution observational datasets of global precipitation and land cover to examine how land cover changes in response to prolonged drought events worldwide over the past three decades. Our findings show a strong relationship between the occurrence of prolonged droughts and the subsequent changes in land cover, as in Fig. 1. This correlation shows variability across different regions, with a generally stronger association observed in areas affected by drought compared to those unaffected, emphasizing the significant impact of drought on land cover dynamics (Fig. 3). Moreover, we found that low-income countries exhibit the most pronounced trends in land cover change following prolonged droughts, whereas high-income countries show the least variation. Attributing these changes solely to drought can be misleading, as various factors, such as socio-economic conditions, political regime shifts and land management practices, have been identified as driving forces behind land cover alterations^14,33^. However, findings from our study at global level stress out the important role that droughts have on land cover transformation.

Drought events are significant drivers of land degradation, amplifying the vulnerability of ecosystems and communities across the globe. This study confirms that droughts contribute to land cover changes, potentially affecting agricultural productivity, ecosystems, and socio-economic stability. The results above highlight global and regional patterns of land cover change in response to drought, emphasizing how droughts can exacerbate existing vulnerabilities, especially in low-income and developing economies. The findings align with previous research at local level, indicating that droughts can lead to land degradation, reduced crop yields, and increased vulnerability for communities, particularly in rural and agricultural sectors^14^.

### Impact of drought on land cover change

As the study demonstrates, droughts lead to changes in land cover globally, with noticeable effects in different regions based on their specific socio-economic contexts^40,41^. The reduction in shrub/grassland and bare areas following prolonged droughts, observed both globally and across different economic strata, as shown in Fig. 4, aligns with the expected pattern of land degradation under drought conditions^42–44^. Specifically, shrubs and grasslands showed significant decreases in many developing economies, which is consistent with previous research suggesting that drought impacts are particularly pronounced in areas reliant on natural grasslands for grazing and agricultural production^14,45^. As also demonstrated in Erb et al.^41^agricultural systems in regions susceptible to drought often face a greater threat of land degradation. In contrast, as shown in Figs. 2 and 5, high-income countries experienced fewer dramatic changes in these land cover types, possibly due to more effective management practices and more stable agricultural systems, although a marked decrease in shrubland and grassland was recorded in 2005. This observation emphasises the need for a broader understanding of drought impacts beyond just low-income regions^40,46^.

The study corroborates existing literature that droughts degrade land in various ways, including soil erosion and desertification, which can further exacerbate drought conditions^10^.

Severe water deficits affect vegetation from the leaf scale upward. Stomatal closure lowers photosynthesis and transpiration; when soil moisture remains below the wilting point, xylem embolism and carbon starvation trigger widespread woody mortality in both temperate and tropical biomes^23,27^. Satellite observations show that such physiological stress reduced global terrestrial net primary production by ~ 1–2% during extreme droughts^25^. On the other hand, canopy loss increase surface albedo and reduces aerodynamic roughness, weakening turbulent moisture fluxes; this positive feedback intensifies near-surface aridity and can double the atmospheric residence during droughts^26,43^. Desiccated fine fuels increase wildfire frequency, while weakened trees promote pathogen outbreaks, compounding mortality^23,28^. Model experiments for the Sahel demonstrate that land–atmosphere feedbacks accelerate dryland expansion, effectively shifting biome boundaries toward more arid equilibria^47^.

Where drought co-occurs with deforestation, as in the south-eastern Amazon, regional moisture recycling has declined by up to one-third, creating a self-reinforcing “drought-deforestation” loop that is pushing forest patches toward a savanna regime^10,29^. Post-drought recovery studies further show that ecosystems with high initial canopy loss require two- to four-times longer to regain pre-drought productivity, highlighting the risk of crossing resilience thresholds^48^. These coupled eco-hydrological processes explain the pronounced decreases in shrub/grassland and bare areas that we observe after multi-year droughts and underscore why mechanistic understanding is essential for attributing land-cover change to climate variability. For instance, reductions in tree cover and increases in cropland in certain regions, shown in Fig. 4, indicate that anthropogenic activities, such as agricultural expansion, continue to shape land cover changes^41,45,47^. As also demonstrated in Geist and Lambin^44^deforestation linked to agricultural expansion often reduces the land’s ability to retain moisture, magnifying drought effects. These findings reflect a dual feedback loop where agricultural expansion contributes to land degradation, and drought exacerbates the challenges posed by such land-use practices. In particular, the deforestation associated with agricultural expansion in developing economies can reduce the land’s ability to retain moisture, thereby increasing the severity and duration of droughts^10^. As also noted in Fensholt et al.^40^when vegetation cover declines, soils become more vulnerable to erosion under drought stress. The observed trend of increasing tree cover in recent years, particularly in some developed economies as shown in Figs. 4 and 5, may indicate a shift towards reforestation or a reduction in the rate of deforestation^45,49^.

Land-cover outcomes emerge not only from biophysical stress due to severe droughts but also from demographic pressure, market incentives and governance capacity. Rapid population growth and rising food demand in low-income nations have driven cropland expansion into woody savannas and wetlands, quadrupling the global rate of conversion since the 1990s^22,50^. Global commodity markets and foreign land acquisitions directed capital toward large-scale plantations, accelerating deforestation fronts in Latin America and Southeast Asia^51^. On the other hand, livelihood reduction during drought can lead to rural-to-urban migration, with the consequent abandonment of marginal fields enabling woody encroachment or shrub re-establishment, a process frequently misinterpreted as ecological recovery^14,52^. In high-income settings, technological buffering and subsidy schemes decouple land-cover responses from drought, shifting change toward peri-urban sealing and bioenergy crops^45,53^. Recognising these socio-economic pathways is critical for designing adaptation policies that do not externalise drought risk onto vulnerable landscapes.

### Regional variability

The distribution of land cover change is not homogeneous, and results from this study highlight significant spatial and temporal variations, consistent with previous studies that emphasised the disparity in land cover changes between the Global North and South^33,54^. The results reveal that in emerging and developing economies, land cover changes are predominantly driven by agricultural expansion and deforestation, while in advanced economies, land cover changes are more complex and often involve multiple factors such as urbanisation, agricultural intensification, and forest conservation efforts^50^. As also demonstrated in Foley et al.^45^these drivers often have compounding effects on regional land degradation. In many developing economies, the changes in land cover are closely tied to the shift from tree cover, shrub, and grassland to cropland and urban areas, suggesting that economic development, population growth, and urbanisation exacerbate the negative effects of droughts^51^.

Similar to our study, recent satellite-derived vegetation indices show that the physical imprint of prolonged droughts differs sharply across biomes. In semi-arid savannas of the Sahel and central Australia, repeat multi-year droughts reduce herbaceous biomass, expose bare soil and intensify surface heating; the resulting land–atmosphere feedback accelerates woody mortality and expands bare-ground patches by up to 15% within a decade^23,26^. Humid tropical forests usually experience a single dry year, but consecutive droughts can heighten fire susceptibility, pushing forest fragments toward a savanna state^10^. Mid-latitude temperate forests respond mainly through canopy thinning and understory desiccation, creating fuel ladders that facilitated the stand-replacing fires like after the 2003 European heat-drought compound event^35^. Figure 2 shows that in high-income countries the lack of a clear pattern of land cover change during drought events could reflect the greater resilience and adaptive capacity of these regions^48^. The diversified economies and well-established infrastructure in developed economies may buffer the worst effects of drought, allowing for more flexible land management practices. As also indicated in Venter et al.^46^resource availability and policy interventions in developed areas can mitigate drought impacts. However, the trend toward urbanisation and agricultural intensification in these regions warrants attention, as it can have long-term impacts on land cover, particularly if urban sprawl exacerbates the urban heat island effect and leads to increased water consumption, further straining available resources during drought periods^55^.

On the other hand, in low-income and tropical regions, land cover change linked to shrubs and grasslands shows a more direct link to drought impacts, as in Fig. 4, where reduced vegetation cover due to droughts leads to land degradation and worsens food insecurity^52^. As also found in Knapp and Smith^56^grassland productivity is especially sensitive to variations in precipitation, making these ecosystems more vulnerable to drought. The results suggest that agricultural land in these regions is more vulnerable to the effects of drought, with cropland expansion exacerbating the degradation of ecosystems and the availability of grazing land. In particular, shrubs and grasslands, essential for pastoral livelihoods, decrease as drought intensifies, reinforcing the cyclical nature of land degradation and vulnerability to future drought events.

Additionally, Fig. 2 highlights that tropical and arid regions show higher trends in land cover changes, while continental climates tend to show lower variation in land cover change^57^. Climate areas also play a role in how different land cover types react to drought, with shrub/grassland cover reducing in tropical, arid, and temperate climates but increasing in continental regions. As also demonstrated in Wang and Eltahir^47^arid and semi-arid ecosystems can develop strong feedback loops between drought and vegetation loss. Tree cover, on the other hand, is more affected in arid and temperate climates, showing a greater coefficient of variation in these areas^58^. Thus, both income levels and climate conditions significantly shape the extent and type of land cover changes that occur after drought periods, as in Fig. 4, with more pronounced impacts in lower-income and tropical or arid regions^53^.

### Strategies for mitigation and adaptation

The study underscores the importance of implementing sustainable land management practices to enhance resilience to drought and mitigate the adverse effects of land cover changes. While these changes are inevitable under a shifting climate, the pace and magnitude of change can be moderated through the adoption of sustainable management practices^54^. For instance, in Ethiopia, the shift from a solely pastoral lifestyle to an integrated crop-livestock system has effectively reduced vulnerability and enhanced livelihood sustainability in the face of recurrent droughts^31,59^. Similarly, in Vietnam, the transition from monocultural farming to crop rotation has been shown to promote soil revegetation and stabilisation of bare soils^30^. Van Khuc et al.^60^ highlighted that tailored interventions are crucial for mitigating the negative impacts of deforestation and forest degradation. These adaptive strategies are critical in increasing global food security and improving resilience against the adverse effects of drought.

These strategies as well as land management practices must remain flexible and adaptable as climate conditions continue to evolve, and strategies considered today might become insufficient in the future^54^. What works today may not be sufficient in the future, particularly as droughts become more frequent and severe. Therefore, adaptive land management policies must be dynamic and responsive to changing environmental conditions, emphasising resilience and sustainability across various sectors.

### Future research directions

Our study investigated the relationship between global drought events and land cover changes, using correlation analysis as a preliminary descriptive method. While this approach offers initial global insights, it cannot determine causality or the direction of the observed relationships. To deepen understanding, future research should adopt causal inference methods to investigate whether land cover changes are a result of drought or a contributing factor. This is particularly relevant given growing evidence that land cover can influence drought occurrence and intensity.

We also stress the importance of analyzing drought impacts over multiple accumulation times, as the effects vary with duration and intensity. Short-term droughts may affect vegetation health, while prolonged events can lead to permanent land cover changes. Evaluating different timescales and drought accumulation times will clarify land systems’ temporal sensitivity to drought.

Additionally, localized studies are crucial to uncover region-specific drivers and feedbacks, which broader analyses may miss. Future work should integrate socio-economic, political, and land management factors, along with climate and economic projections. Comparative studies and complex system models—such as agent-based^61^ or integrated assessment models—can help identify effective governance structures, economic incentives, and adaptation strategies, while also assessing trade-offs and unintended consequences of future policies.

## Methods

### Data

Four global datasets were exploited to investigate the relationships between drought occurrence and land cover change worldwide at annual scale. We selected global datasets spanning a period of at least 30 years to ensure a representative comparisons^62^. Our analysis is carried out at the global scale and country-based spatial resolution. When assessing the correlation between land cover and non-drought areas, we restricted the analysis to countries with a surface area larger than 100km^2^ to allow a representative spatial assessment of drought condition, resulting in 109 analysed countries.

The TerraClimate dataset was used to calculate the Standardized Precipitation Index (SPI) and assess drought conditions. TerraClimate provides global monthly precipitation data from 1958 to 2023 at a spatial resolution of 1/24°^63^. This dataset integrates station-based observations and uses climatically aided interpolation, incorporating data from sources such as WorldClim, CRU Ts4.0, and JRA55. It is worth noting that other indices such as the self-calibrating Palmer Drought Severity Index (sc-PDSI) and Standardized Precipitation Evapotranspiration Index (SPEI) could have been used to explicitly capture the dynamic of hydro-thermal conditions. However, a number of studies covering Europe^64^East Asia^65^the Mediterranean^66,67^ and Australia^68^showed a high correlation between SPI and those indices when analyzing long drought events, as the moisture balance term (P – PET) that distinguishes SPEI from SPI adds only marginal information in most environments. Temperature-driven evapotranspiration becomes decisive at shorter (monthly to seasonal) windows or in very hot, arid basins, but for assessing the occurrence of year-long droughts the simpler precipitation-only SPI provides similar results than SPEI.

The Copernicus dataset was selected to represent the annual spatial distribution of different land cover types in the period 1992–2022 at the global level^69^. The datasets classify land cover into 22 categories using the United Nations Food and Agriculture Organization’s Land Cover Classification System, leveraging indices like the Normalised Difference Vegetation Index (NDVI) and the Normalised Difference Water Index (NDWI). In this study, the 22 categories were aggregated in 7 main ones, i.e. urban, shrub/grassland, tree cover, cropland, bare areas, sparse vegetation, and other (Supplementary Table 1). The Copernicus dataset has a spatial resolution of 300 m. However, we resampled the dataset at a 1 km resolution to reduce the computational time. Resampling was performed in ArcGIS using the Nearest Neighbour method, which assigns each 1 km cell the value of the 300 m input cell whose center is closest to the center of the output cell. This preserves original land cover categories but may oversimplify heterogeneous areas and potentially overestimate land use change due to reduced spatial detail.

The countries are further classified by income-levels, to account for possible wealth effects, and by climate areas, following the Köppen-Geiger climate classification^70^. The classification of income is performed following the 2019 income division as used by the World Bank^71^.

### Drought analysis

The process included the identification of drought events at grid level and the consequent assessment of the area affected by drought. Firstly, the Standardized Precipitation Index (SPI), a widely adopted drought index, was used to identify drought periods. The SPI is calculated by aggregating precipitation over a chosen accumulation period, comparing it to the long-term record for the same period, fitting it to a probability distribution, and standardising the result^72^. Negative SPI values represent dry conditions, while positive values indicate wet conditions. Short accumulation periods (1–3 months) are usually selected to detect seasonal changes in precipitation patterns, while longer periods (12–48 months) are useful to identify prolonged dry or wet periods^73^. In this study, we selected a 12-months accumulation time as we were interested in assessing the land cover change after the occurrence of a prolonged drought periods. Secondly, once the time series of SPI values were known, we identified drought periods as the years characterized by SPI lower than − 1^74^. This allowed us to delineate the annual gridded areas affected by drought.

### Land cover change analysis

**Fig. 6:**
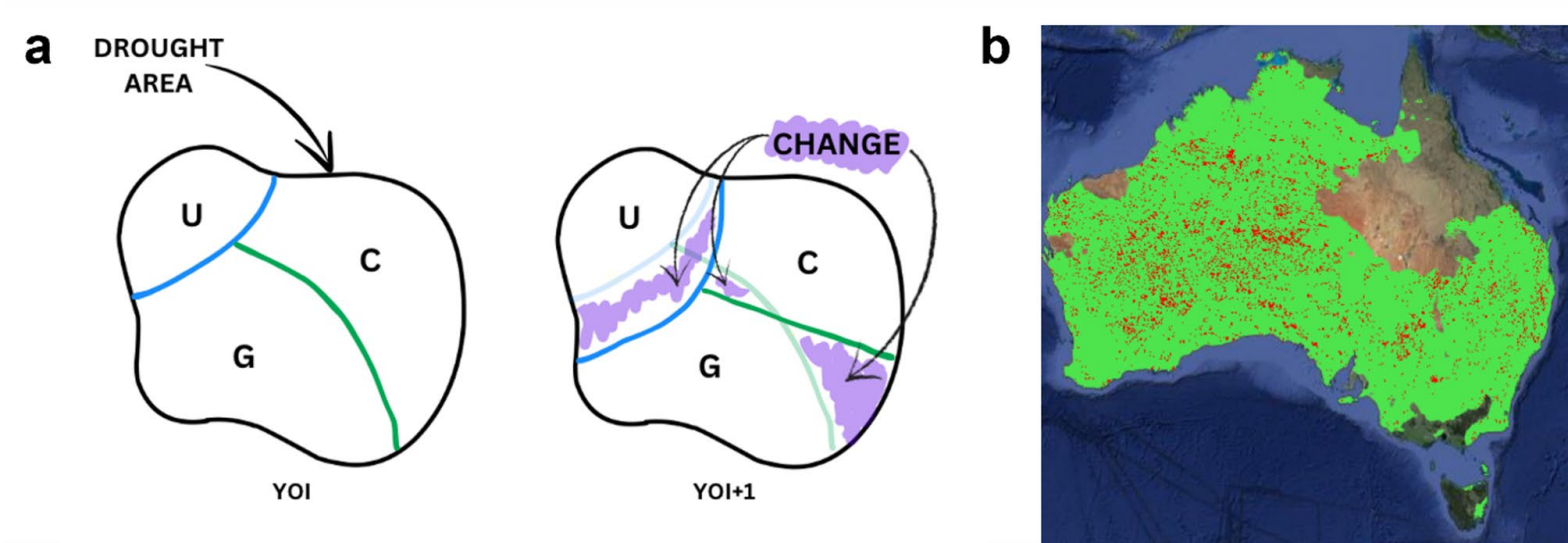
Method used to assess land cover change after a drought. **a** Schematic representation of identifying drought and land cover change. **b** Changes of land cover in Australia for 2019, with green representing no change, while red indicates change.

The gridded information of the drought-affected areas was overlayed with the land cover dataset to create a composite layer. In particular, assuming a specific year of interest YOI, we calculated both the land cover within the drought area at YOI, and the land cover at time YOI *+ 1* considering the drought area extension at YOI. This allowed us to assess the effect of a prolonged drought at YOI on the land cover change in the subsequent year. This approach was selected based on Ceola et al.^75^ and was performed for all land cover categories, for each year, and country. The decision to limit the evaluation of land use change to YOI + 1 was based on the assumption that significant shifts in land cover are more likely to occur shortly after a drought event. Extending the analysis over longer periods introduces greater uncertainty, as other external factors could influence land use patterns over time. By concentrating on the immediate year following the drought (YOI + 1), we sought to isolate changes directly linked to the conditions and processes specific to that period, thereby reducing the impact of unrelated variables. An illustrative example of changes of land cover from YOI to YOI *+ 1* is reported in Fig. 6a, where purple areas indicate alterations in land cover not consistent between the two assessed years. Areas where land cover for the YOI differed from that of YOI + 1 were categorised as “change” (red colour in Fig. 6b). On the other hand, areas within the drought-affected zone where land cover remained consistent between the two years were classified as “no change” (green colour in Fig. 6b). Land cover change data were than aggregated at country level. This analysis was performed merging all land covers in one main class, as well as by assessing changes for the seven different types of land covers previously described.

Finally, the same procedure was followed to assess land cover change outside the drought area (i.e. areas with SPI greater than − 1). Comparing land cover changes within and outside the drought areas can provide a potential correlation between drought occurrence and consequent land cover change.

### Statistical analysis

A trend analysis was performed to investigate the annual variation of land cover change and drought areas. A normalisation based on Z-score approach was performed over the 1992–2021 period to compare the results from countries with different extent, income levels, and climate characteristics. Then, we assessed the correlation between drought areas and land cover changes using a pair-wise Spearman correlation coefficients and p-values for the 109 countries.

The objective of this analysis was to explore the relationship between land cover changes within and outside the drought areas and better understand how drought events can influence landscape changes. Also in this case, the correlation results are classified among countries with the same income level and climate classification. The correlation analysis was performed only with the combined land cover classes. The variation coefficient for the different land covers was calculated as the ratio between the standard deviation and the mean in the 1992–2021 period to explore the response of the different land cover changes to prolonged drought periods.

## Supplementary Information

Below is the link to the electronic supplementary material.

Supplementary Material 1

## Data Availability

The Copernicus dataset was selected to represent the annual spatial distribution of different land cover types in the period 1992–2022 at the global level. The dataset is publicly available at: https://cds.climate.copernicus.eu/datasets/satellite-land-cover?tab=overview. The TerraClimate dataset was used to calculate the Standardized Precipitation Index (SPI) and assess drought conditions. The TerraClimate dataset is publicly available at https://www.climatologylab.org/terraclimate.html.
