# Supplementary material for: Exploring land cover change after prolonged droughts at the global level
Source: Sci Rep. 2025 Aug 5;15:28520. doi: 10.1038/s41598-025-14713-6 (PMC12325978; doi:10.1038/s41598-025-14713-6)
Supplement: Supplementary file 1 — Supplementary Material 1 [file 41598_2025_14713_MOESM1_ESM.docx]

Supplementary material for

**Exploring land cover change after prolonged droughts at the global level**

Felicia Engman^1,*^, Ester Kortekaas^1,*^, Luigia Brandimarte^1^, Maurizio Mazzoleni^2,3^

^1^ Royal Institute of Technology, KTH, Department of Sustainable Development, Environmental Science and Engineering, Stockholm, Sweden

^2^ Institute for Environmental Studies, Vrije Universiteit Amsterdam, Amsterdam, The Netherlands

^3^ Karolinska Institutet, Department of Global Public Health, Stockholm, Sweden

* These authors have equally contributed

Corresponding author: Luigia Brandimarte, luigia.brandimarte@abe.kth.se

#

# This document includes

Figure 1 to 2

Table 1


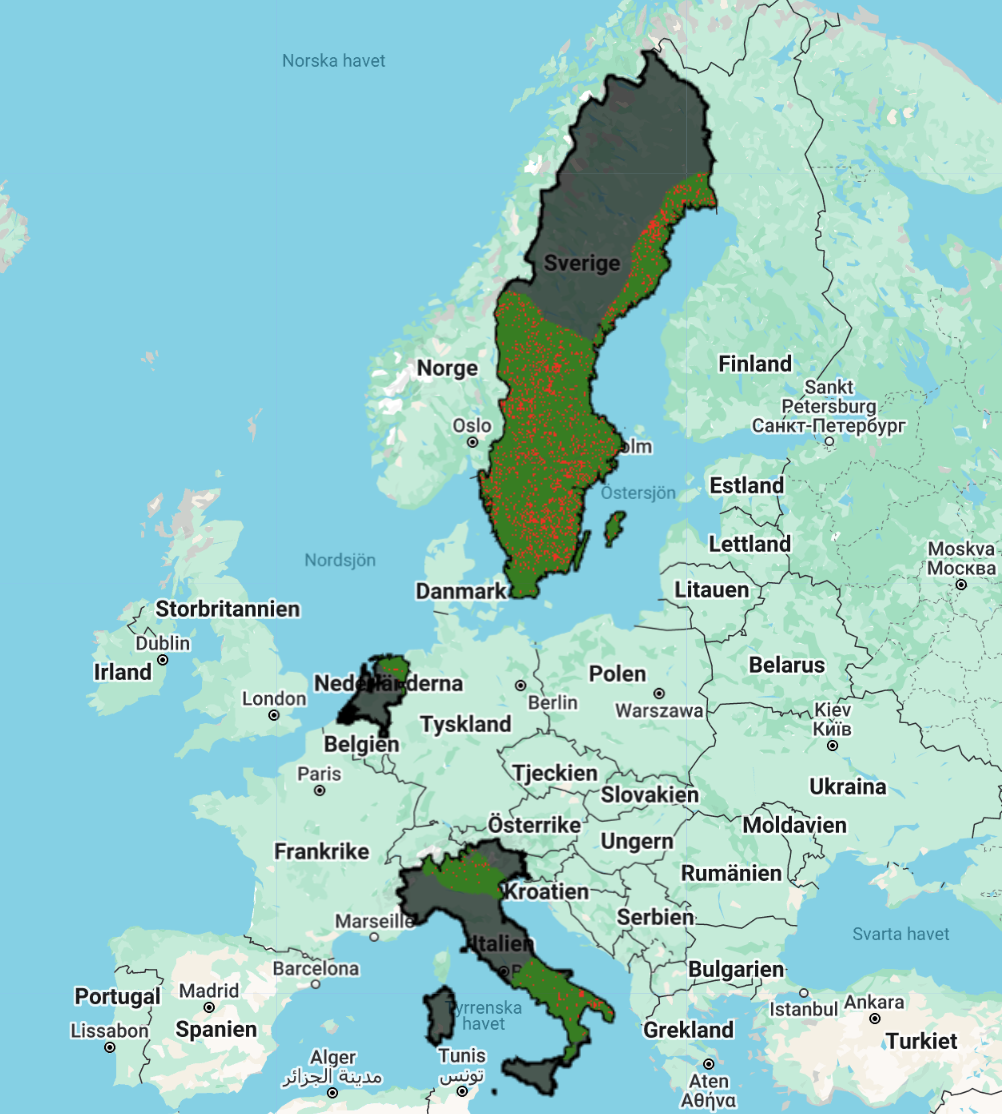


**Supplementary Fig. 1|** Example of the gridded analysis performed in this study for a few countries in Europe


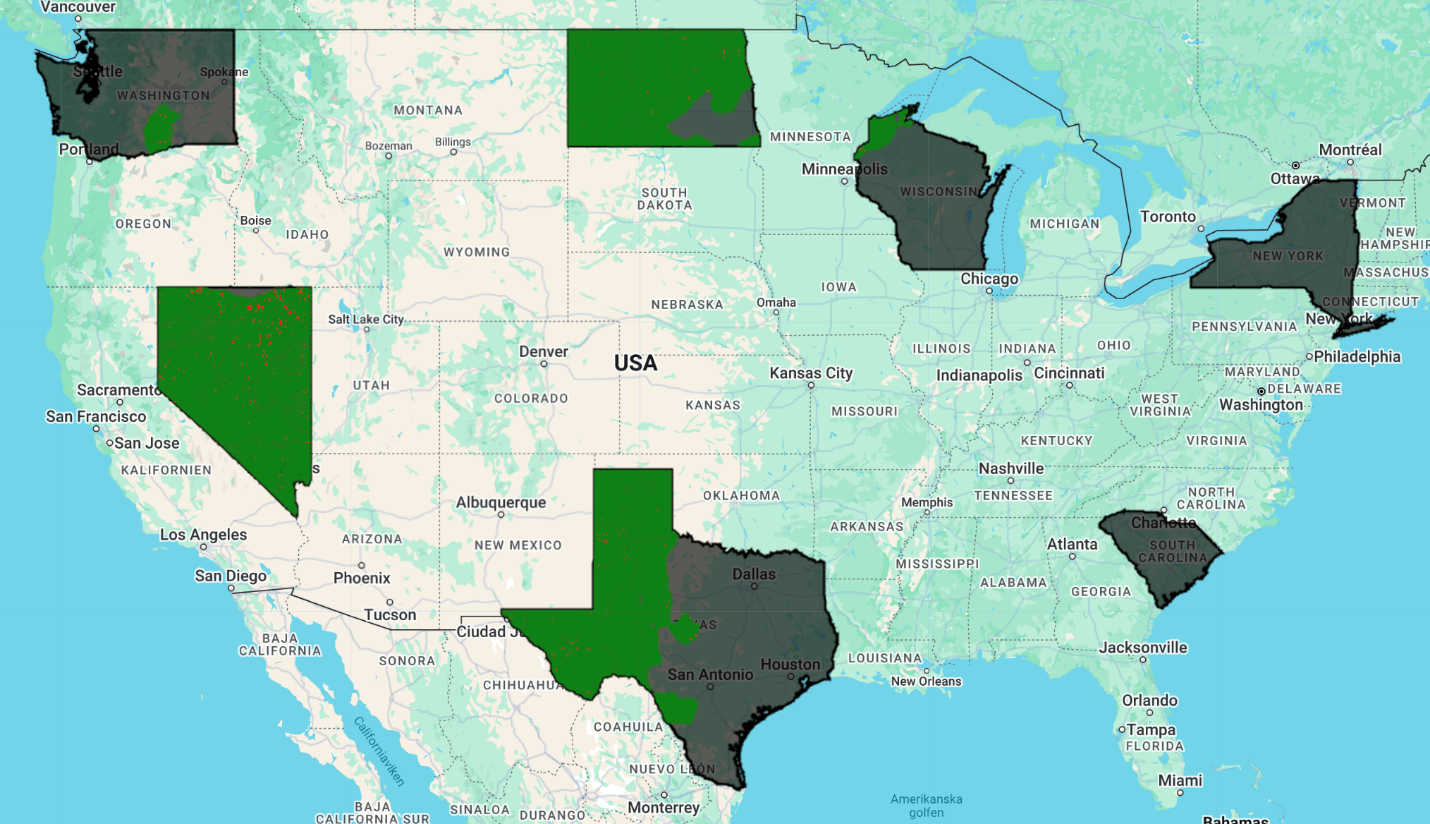


**Supplementary Fig. 2|** Example of the gridded analysis performed in this study for a few States in the United States of America

**Supplementary Table 1**| Aggregated land cover classifications

| **Aggregated classifications** | **Original classifications** |
| --- | --- |
| Urban areas | Urban areas |
| Shrub- and grassland | Shrubland |
|  | Evergreen shrubland |
|  | Decidous shrubland |
|  | Grassland |
| Tree cover | Tree cover, broadleaved, evergreen, closed to open (>15%) |
|  | Tree cover, broadleaved, deciduous, closed to open (>15%) |
|  | Tree cover, broadleaved, deciduous, closed (>40%) |
|  | Tree cover, broadleaved, deciduous, open (15-40%) |
|  | Tree cover, needleleaved, evergreen, closed to open (>15%) |
|  | Tree cover, needleleaved, evergreen, closed (>40%) |
|  | Tree cover, needleleaved, evergreen, open (15- 40%) |
|  | Tree cover, needleleaved, deciduous, closed to open (>15%) |
|  | Tree cover, needleleaved, deciduous, open (15-40%) |
|  | Tree cover, needleleaved, deciduous, closed to open (>15%) |
|  | Tree cover, mixed leaf type (broadleaved and needle leaved) |
| Cropland | Cropland, rainfed |
|  | Cropland, rainfed, herbaceous cover |
|  | Cropland, rainfed, tree, or shrub cover |
|  | Cropland, irrigated or post-flooding |
| Bare areas | Bare areas |
|  | Consolidated bare areas |
|  | Unconsolidated bare areas |
| Sparse vegetation | Sparse vegetation (tree, shrub, herbaceous cover) (<15%) |
|  | Sparse tree (<15%) |
|  | Sparse shrub (<15%) |
|  | Sparse herbaceous cover (<15%) |
| Other | Lichens and mosses |
|  | Tree cover, flooded, fresh or brackish water |
|  | Tree cover, flooded, saline water |
|  | Mosaic natural vegetation (>50%) / cropland (<50%) |
|  | Mosaic tree and shrub (>50%) / herbaceous cover (<50%) |
|  | Mosaic herbaceous cover (>50%) / tree and shrub (<50%) |
|  | Shrub or herbaceous cover, flooded, fresh/saline/brackish water |
|  | Water bodies |
|  | Permanent snow and ice |
|  | No data |
